# A Case-Control Study on Chromosomal Anomalies in Parents Experiencing Repeated Spontaneous Abortions From Northern India

**DOI:** 10.7759/cureus.19819

**Published:** 2021-11-22

**Authors:** Malamoni Dutta, Putul Mahanta, Bharati Basumatary, Ranjumoni Konwar

**Affiliations:** 1 Anatomy, Assam Medical College, Dibrugarh, IND; 2 Forensic Medicine and Toxicology, Assam Medical College and Hospital, Dibrugarh, IND; 3 Radiology, Fakhruddin Ali Ahmed Medical College (FAAMC) and Hospital, Barpeta, IND

**Keywords:** spontaneous abortion, numerical chromosomal defects, robertsonian translocation, structural chromosomal aberrations, repeated pregnancy loss

## Abstract

Objectives

Many women lose their fetuses through miscarriage due to a variety of causes. The incidence of three or more consecutive pregnancy losses is often classified as repeated spontaneous abortion (RSA) and is considered the most frustrating and complex area in reproductive medicine. Parental chromosomal abnormalities, underlying medical condition, heritable or acquired thrombophilias, immunologic abnormalities, infections, and environmental factors are reported to be possible etiologies responsible for RSA. Gametes with unbalanced chromosomes, which are formed when abnormalities exist in parent chromosomes, are one such cause and are responsible for about 50-60% of first-trimester pregnancy loss. This paper aims to identify whether there is an association between chromosomal anomalies in parents and RSA.

Method

A case-control study was performed on a total sample size of 600 individuals, including 150 couples with a history of RSA and 150 fertile couples as control. The participants were cytogenetically analyzed using G-banding. Associations between variables were tested using Chi-square and Fisher’s exact test (a p-value<0.05 was considered significant). Informed consent from participants and institutional ethical clearance was obtained before the research began.

Results

Chromosomal anomalies were detected in 21 individuals (7%) with a history of RSA. Female preponderance was observed with a female to male ratio of 2.5:1. Structural chromosomal aberrations (SCAs) were detected in 17 patients, with nine (53%) cases showing balanced reciprocal translocation (involving chromosomes 1,3,6,8,12,13,15,16,18,22 and X) and three (17.65%) cases of Robertsonian translocation (exclusively in males). Mosaicism was observed in four (19.05%) cases. A statistically significant positive association (p-value <0.05) was observed between the presence of parental chromosomal anomalies and RSA.

Conclusion

These results support an association between RSA and parental chromosomal abnormalities. Currently, clinicians treating cases of RSA face challenging clinical conditions. Identifying a cytogenetic cause for RSA may be of great help to clinicians who manage affected couples.

## Introduction

The definition of repeated spontaneous abortions (RSA) is varied and affects 2-5% of women. Cytogenetic abnormalities are one of the more common causes of RSA [1]. Parents with anomalous karyotypes contribute to either infertility or abortions [2,3]. However, research has shown that parents experiencing abortion may possess chromosomal abnormalities in one or both partners [2]. Studies show that 50-60% of pregnancy loss during the first trimester resulted from chromosomal abnormalities, which can be of the parental origin or arise de novo during embryonic development despite both parents having normal chromosomes [4,5].

About 30% of pregnancy losses take place as soon as implantation occurs [6]. One-fourth of pregnancies are aborted before 14 weeks and are diagnosed clinically, but the intrauterine period of six to eight weeks is considered crucial. About 20% of fetuses aborted during this period show chromosomal abnormality (CA). Fetal aneuploidy is found in approximately 90% of zero to six weeks pregnancy losses, 50% in sporadic losses, and 30% in losses occurring between 16 and 19 weeks of pregnancy [7]. The most common chromosomes to show aneuploidy in early pregnancy abortions are chromosomes 13, 18, 21, X, and Y, mainly resulting from parental carriership [8-10].

In one study, 7.6% of patients with RSA history were diagnosed with numerical or structural chromosomal abnormalities. The same study also observed a high rate of translocations (46%) [3]. As revealed in some studies, this fetal genomic incompatibility with life was associated with chromosomal aneuploidies and mosaicism within the embryo or abortus [11,12]. For cases where the parents have a history of RSA, prenatal diagnosis during the 16th week of gestation is strongly recommended [13] as structural rearrangement is often associated with RSA cases [14]. However, a comprehensive chromosome screening known as preimplantation genetic screening should be performed to determine which embryos are euploid so that only healthy embryos are implanted [15].

Although various studies have been undertaken around the globe to assess the impact of parental chromosomal abnormalities in RSA, limited data are available from the Northern Indian context. Therefore, the current study was undertaken to evaluate the chromosomal aberrations in parents experiencing RSA from Northern India to enable clinicians to manage RSA better. To that end, the present study assesses the association between RSA and CA in parents with the following null hypothesis: H0 - recurrent spontaneous abortion is not associated with the chromosomal abnormalities of the parents.

## Materials and methods

This case-control research was performed from September 2015 to October 2018 and included 150 couples experiencing RSA (case group) and 150 fertile couples (control group) in the reproductive age range of 19-48 years. The participants in both groups were ethnicity and age-matched and were selected randomly from the Northern part of India. Couples with a history of two or more consecutive pregnancy losses before 20 weeks gestation in the absence of any apparent cause were included as cases in the study. At the same time, the control group had fertile couples without any history of spontaneous abortion. Patients with known immunological, infectious, or endocrinal disorders, or those who did not consent to participate in the study, were excluded.

For the genomic study, 5 ml of peripheral blood was taken from each participant (in both case and control groups), and the lymphocyte culture technique was used for karyotyping [16]. The blood was collected in a heparinized vial, and cultures were harvested. Metaphase spreads were made from phytohemagglutinin-stimulated peripheral lymphocytes using standard cytogenetic techniques [17]. Karyotype was prepared using the G-banding method with trypsin and Giemsa staining (GTG). G-banding produces a series of dark and light bands that allow for the identification of each chromosome. For each patient, a minimum of 30 metaphases was examined under the microscope. Chromosomes were analyzed with the help of Cyto-vision software (Applied Imaging, Rochester, NY, USA). Chromosome identification was made following the International System for Human Cytogenomic Nomenclature [18].

Before collecting the samples, ethical approval from the ethics committee was obtained from Gauhati Medical College, Guwahati vide Ref No. MC-233/2013/155. Written informed consent was also obtained from the participants before data and sample collection.

Data were analyzed using Microsoft Excel (Microsoft Corporation, Redmond, WA) and the Statistical Package for the Social Sciences version 20 (IBM Corp., Armonk, New York). A P-value <0.05 was considered significant. The Chi-square test or Fisher's exact test was employed to determine whether there was an association between RSA and chromosomal anomalies in the parents. The relative occurrence of different chromosomal anomalies among the study participants and the extent of contribution of a parent’s CA to RSA is presented in the current study.

## Results

A total of 21 (7%) individuals out of an overall sample size of 600 had chromosomal anomalies, and all individuals were from the group of parents with a history of RSA. In the control group, all participants showed a normal karyotype. Of those 21 individuals with abnormalities, 15 (71.43%) were female, and 6 (28.57%) were male, a 2.5:1 female-to-male ratio. Among the 21 individuals with an abnormal karyotype, the mean maternal age was 30.13 years, and the mean paternal age was 32.17 years. No consanguineous marriages were reported among the studied couples.

Numerical chromosomal aberrations were found in 4 (1.33%) cases, and structural chromosomal aberrations (SCA) were observed in 17 (5.7%) cases. The different chromosomal anomalies consisted of balanced reciprocal translocations (n=9), Robertsonian translocations (n=3), deletions (n=2), inversions (n=3), and numerical aberrations (n=4).

Structural chromosomal aberrations

Table 1 shows the different types of SCA cases, along with age and sex reported under various categories. Out of 17 SCAs, 9 (53%) cases showed balanced reciprocal translocation, which involved chromosomes 1, 3, 6, 8, 12, 13, 15, 16, 18, 22, and X. The result highlight that all complicated cases of reciprocal translocations occurred in females.

**Table 1:** Types of structural chromosomal anomalies

Structural anomalies	Age	Sex
(A) Reciprocal translocation		
46,XX, t(6;18)(q27;q23)	22	F
46,XX, t(1;3)(q43;q29)	30	F
46,XX, t(18;22)(q21;q12)	29	F
46,XX, t(8;22)(p21;q13.1)	25	F
46,XX, t(X;16)(q28;q24)	30	F
46,XX, t(13;15)(p11.2;q22.1)	32	F
46,XX, t(X;1)(q21.1;q32)	27	F
46,XX, t(6;16)(p12;q24)	34	F
46,XX, t(12;15)(q15;q25)	37	F
(B) Robertsonian translocation		
45,XY,rob(13;14)(q10;q10)	35	M
45,XY, rob(14;15)(q10;q10)	32	M
45,XY, rob(13;14)(q10;q10)	33	M
(C) Deletion		
46,XY, del(10)(p14)	30	M
46X,(del)X(p21-pter)	29	F
(D) Inversion		
46,XX, inv(16)(p13;q22)	36	F
46,XX, inv(9)(p11;q13)	25	F
46,XY, inv(9)(p11;q13)	27	M

**Figure 1:**
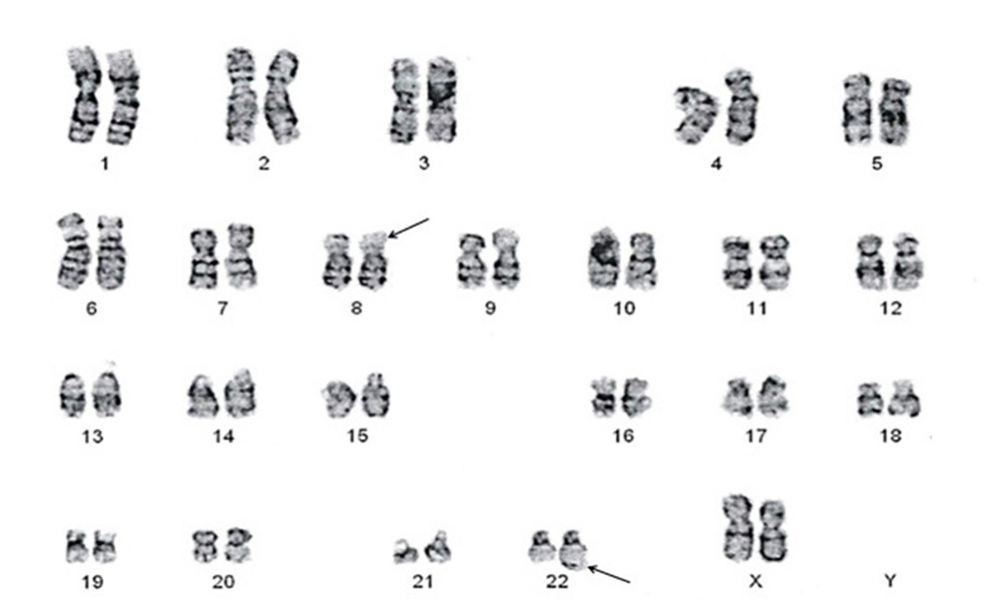
Translocation between chromosomes 8 and 22

The translocation between chromosomes 8 and 22 in a female patient is shown in Figure 1.

**Figure 2:**
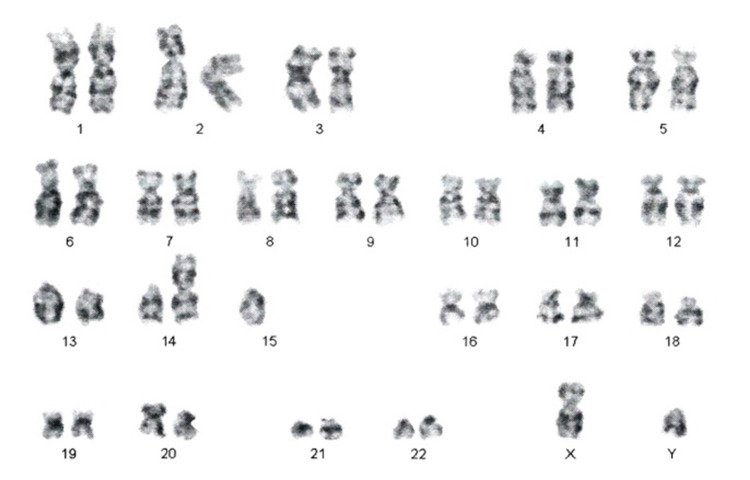
Robertsonian translocation between chromosomes 14 and 15

Out of 17 SCAs, three (17.65%) individuals (all male) had Robertsonian translocations involving chromosomes 13, 14, and 15. Figure 2 shows a Robertsonian translocation between chromosomes 14 and 15.

Only two out of 17 SCAs (11.76%) showed partial deletion. One participant had a partial deletion of the terminal portion of the short arm of chromosome 10. In the other case, the deletion was detected in the short arm of chromosome X. Furthermore, only three cases out of 17 SCAs (17.65%) showed an inversion. One participant had a pericentric inversion in chromosome 16. In the other two patients, a pericentric inversion was observed on chromosome 9.

Numerical chromosomal aberrations

**Figure 3:**
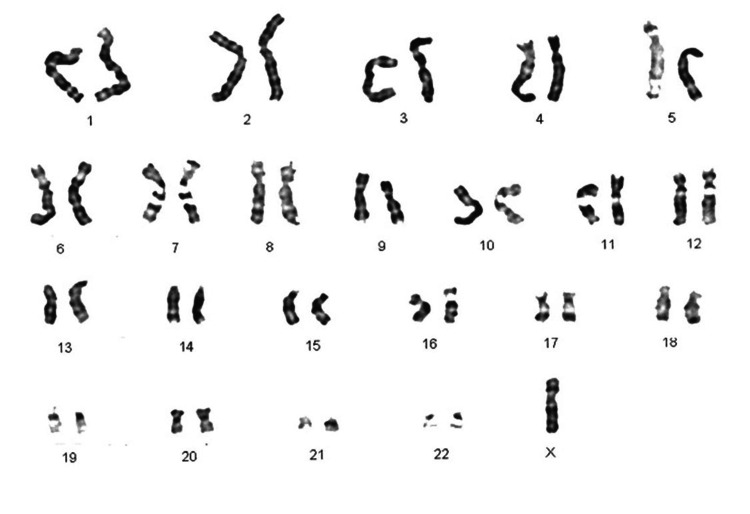
Karyotype of a female case with 45X chromosome

None of the numerical chromosomal anomalies showed pure karyotypes in the present survey. Out of 21 cases with chromosomal aberrations, four (19.05%) were mosaics. Two females were mosaics with monosomy (45X) (Figure 3).

A single female was mosaic with 47, XXX, and one male was mosaic with 47, XXY. Fisher’s exact test showed that a significant association exists between RSA and the father's CA, indicating that the CA of the father may be related to the occurrence of RSA (Table 2).

**Table 2:** Association of RSA and father’s CA as tested by Fisher’s exact test RSA: repeated spontaneous abortion, CA: chromosomal abnormality.

		CA in father		P-value
		Absent	Present	
History of RSA in tested couples	No (n=150)	150	0	0.03
	Yes (n=150)	144	6	
Total (n=300)		294	6	

A Chi-square test revealed a significant association between the presence of CA in the mother and RSA (p-value <0.001; Table 3).

**Table 3:** RSA and CA in the mother as tested by Chi-square ^##^Statistically significant. RSA: repeated spontaneous abortion, CA: chromosomal abnormality.

		CA in mother		p-value^##^
		Absent	Present	
History of RSA in tested couples	No (n=150)	150	0	<0.001
	Yes (n=150)	135	15	
Total (n=300)		285	15	

Hence, a CA present in either parent may be a cause of RSA.

## Discussion

In the present study, significant differences in CA were found while comparing couples with RSA to controls, an association that is consistent with previous studies [11,19]. The variations in frequencies of CA occurrence reported by different studies may, therefore, be attributed to sampling biases such as different sample sizes and disparities in sample selection.

The current study's data suggest that balanced reciprocal translocations (which occurred in nine females) occurring in either parent may be a cause of RSA, a result that is consistent with previous research [20]. In support of this conclusion, previous research uncovered an association between such chromosomal anomalies with other gynecological complications like sterility, RSA, and malformed offspring [21]. A common age in carriers of these translocations is less than 35 years [22]. In another study, a higher prevalence of balanced chromosomal rearrangements among females was also noted [3].

Three male individuals in the current study had Robertsonian translocations, which resulted from the fusion of the long arms of chromosomes 13, 14, or 15. Although all human acrocentric chromosomes, i.e., chromosomes 13, 14, 15, 21, and 22, can participate in Robertsonian translocations chromosomes 13 and 14 are frequently involved constituting almost 85% of all Robertsonian translocations [23,24]. However, in our study, chromosome 15 was not involved, though it is a common occurrence.

Of the three cases of inversion, two were in females (chromosomes 9 and 16) and one in a male (chromosome 9). Pericentric inversion of chromosome 9 is one of the most prevalent structural balanced chromosome rearrangements. Although it does not appear to be linked to atypical phenotypes, numerous conflicting studies have suggested that it may cause anomalous clinical problems such as infertility and recurrent abortions [25,26]. All individuals with inversions in this study were phenotypically normal. Balanced inversions, though, do not confer a phenotypic effect in most cases, miscarriage and chromosomally unbalanced gametes can be observed in some cases [27].

The occurrence of an inversion on chromosome 16 in a female with RSA is consistent with results from a recent study [25]. The same study also reported the deletion of a portion of chromosome 10 in a male partner. Previous studies have also reported trisomy 16 as the most observed trisomy in early pregnancy loss [28]. The above results report an association of RSA with CA.

The deletion of the terminal portion of the short arm of chromosome X in a female partner reflects a trend discussed previously [29]. Although the exact relationship between partial deletions in X chromosomes and RSA is not understood, however, some research studies suggest it as a factor influencing premature ovarian failure among women [27] causing RSA.

All cases with numerical abnormalities found in this study were mosaics. Numerical chromosomal anomalies are less frequent than structural aberrations in the present study, and 2.34% of mosaicism in the current research is similar to results from another study [3]. Women with X-chromosome mosaicism have a poor state of development in the oocytes, which makes them vulnerable to embryonic death [27].

In the present study, no consanguineous marriages were reported among the studied couples. Studies have reported a significant association between RSA and preterm delivery with consanguinity due to an increase in homozygosity of autosomal recessive conditions linked to the pathogenesis of those conditions [30]. Therefore, prior marriage genetic tests and standard karyotyping methods may also be necessary, especially for consanguineous partners.

Limitations

In the present study, karyotyping using the lymphocyte culture method was performed by analyzing G-banded chromosomes. Further studies using more sophisticated technologies such as assay comparative genomic hybridization (aCGH) and Next Generation Sequencing (NGS) may elucidate molecular mechanisms, which were not done in the present study. Another issue to consider is that this study did not discuss potential biases in its sampling or co-factors that may be contributing to the frequency of certain abnormalities or cases of RSA among its study population.

## Conclusions

The correlation of genetic aberrations in either of the parent may be significantly associated with RSA. Parents experiencing RSA should undergo cytogenetic analysis to better inform clinical management approaches. Because RSA can be a challenging clinical entity for gynecologists to manage and can have diverse negative psychological, social, and economic impacts on parents and the health care system, we here reported on the frequency of CA among North Indian parents with a history of RSA. Our results show a promising linkage between CA and RSA, a conclusion supported by other recent studies. Therefore, cytogenetic analysis of couples with a history of RSA may be recommended as a standard procedure. A multidisciplinary and collaborative approach between obstetricians, geneticists, hematologists, scientists, and bioethicists combined with an effective awareness program will contribute to relieving the burden of RSA.
